# *Clonorchis sinensis* Infection prevents DSS-induced Colitis Via Lithocholic Acid in a Gut Microbiota-Dependent Manner

**DOI:** 10.1007/s10753-025-02241-4

**Published:** 2025-02-27

**Authors:** Beibei Zhang, Na Xu, Zheng-Rui Bian, Chen Zhang, Xing Li, Xin-Xin Ren, Zhihua Jiang, Zhongdao Wu, Qian Yu, Kui-Yang Zheng, Mu-Xin Chen, Chao Yan

**Affiliations:** 1 Jiangsu Key Laboratory of Immunity and Metabolism, Department of Pathogenic Biology and Immunology, National Demonstration Center for Experimental Basic Medical Science Education, Laboratory of Infection and Immunity, Xuzhou Medical University, Xuzhou, People’s Republic of China; 2 National Key Laboratory of Intelligent Tracking and Forecasting for Infectious Diseases, National Institute of Parasitic Diseases, Chinese Center for Disease Control and Prevention (Chinese Center for Tropical Diseases Research), Key Laboratory of Parasite and Vector Biology, Ministry of Health, WHO Collaborating Center of Malaria, Schistosomiasis and Filariasis, Shanghai, 200025 People’s Republic of China; 3 Guangxi Key Laboratory for the Prevention and Control of Viral Hepatitis, Guangxi Zhuang Autonomous Region Center for Disease Control and Prevention, Institute of Parasitic Disease Control and Prevention, Nanning, China; 4 Department of Parasitology, Key Laboratory of Tropical Disease Control (Ministry of Education), Zhongshan School of Medicine, Sun Yat-Sen University, Guangzhou, 510080 People’s Republic of China; 5 Hainan Tropical Diseases Research Center (Hainan Sub-Center, Chinese Center for Tropical Diseases Research), Haikou, 571199 People’s Republic of China

**Keywords:** DSS-induced ulcerative colitis, *Clonorchis sinensis*, Intestinal microbiota, Secondary bile acids, TGR5

## Abstract

Increasing evidence demonstrates that helminth and its components can ameliorate ulcerative colitis. *Clonorchis sinensis* (*C. sinensis*) is a kind of helminth that dwells in the bile ducts for many years, but the roles and underlying mechanisms of *C. sinensis*-induced protection from colitis are not elucidated. In the present study, the mice were infected with 50 *C. sinensis* metacercariae and further administrated with 4% Dextran Sodium Sulfate (DSS) in drinking water for 7 days on days 49 post-infection. The disease severity and the integrity of gut barriers were evaluated. Gut microbiota was measured using 16sRNA sequencing, and bile acids in the colon were detected by Liquid Chromatography Mass Spectrometry (LC/MS). The Co-housing approach or microbiota deletion with additional supplies of secondary bile acids (SBAs) was employed to investigate the roles of gut microbiota in the protection from colitis. *C. sinensis* infection moderated the dysbiosis of the intestinal microbiota and increased the levels of SBAs and bile acid receptor Takeda G protein-coupled receptor 5 (TGR5), which finally benefited anti-inflammation and ameliorated the severity of DSS-induced colitis. Co-housing with *C. sinensis*-infected mice, and non-infected mice with colitis also showed an increase of TGR5, decreased pro-inflammatory cytokines, and a reduction in the severity of colitis, compared to those mice suffering from colitis without co-housing. Furthermore, *C. sinensis*-induced protective effects on colitis were attenuated by microbiota deletion, while SBAs (lithocholic acid, LCA) supplementation reversed the colitis. The present study demonstrates that *C. sinensis* infection ameliorates DSS-induced ulcerative colitis in mice, which is dependent on gut microbiota-associated SBAs.

**Supplementary Information:**

The online version contains supplementary material available at 10.1007/s10753-025-02241-4.

## Introduction

Ulcerative colitis (UC) is a chronic, non-specific inflammatory disease characterized by relapsing injuries of the idiopathic epithelial barrier and disruption of inflammation homeostasis in the colon. This disease is prevalent worldwide, with more than 0.3% incidence in Western countries, while there is a dramatic increase in the prevalence of UC in newly industrialized countries such as China [1, 2]. Now, multiple medical therapies are used to mitigate UC. However, there are still several challenges, including the potential for long-term medication side effects, the possibility of relapse following drug withdrawal, and the high cost of treatment [3]. Therefore, the medical demand for the disease is increasing. However, the pathogenesis of UC is still obscure and remains unclear.

Many factors may contribute to this disease, including environment, host genetics, immune homeostasis, and dysbiosis of intestinal microbiota. Accumulating evidence demonstrates that gut microbiota and its metabolite play a crucial role in the maintenance of intestinal homeostasis, and dysbiosis of the intestinal microbiota is considered a key event in the progress of UC[4–6]. Compared with the healthy subject, the diversity of gut microbiota has been decreased and its composition has also been significantly altered in UC patients, showing a reduction in *Bacteroidota* and an increase in *Bacillota* in the gut of UC patients [7]. It was also found that the contents of gut microbiota mediating secondary bile acids (SBAs), such as deoxycholic acid (DCA) and lithocholic acid (LCA) are remarkably reduced in UC patients, and SBAs supplementation reverses the intestinal inflammation in the models of DSS induced-colitis[8]. SBAs can be synthesized from free primary bile acids (PBAs) by gut bacteria with the activities of 7α/β decarboxylase including *Lachnospiraceae* and *Ruminococcaceae* families [9, 10]. In particular, Takeda G protein-coupled receptor 5 (TGR5) acting as the one of most important bile acid receptors mediates SBAs such as DCA and LCA-induced anti-intestinal inflammation [8, 11].

The hygiene hypothesis suggests that the elimination of helminth infection contributes to the increased prevalence of inflammatory diseases in industrialized countries with well-sanitary conditions [12, 13]. Epidemiological evidence also demonstrates a negative correlation between helminth infection and inflammatory disordered diseases[14]. ‘Hygiene hypothesis’ provides a new insight into the treatment of UC, which considers that helminths have potent capacities to regulate the immune responses of their host and infection with helminths ameliorates the severity of colitis in a mouse model of the disease[15], although the mechanisms are largely unknown.

*Clonorchis sinensis* is a liver fluke that dwells in the bile ducts of definitive hosts for a long time (~ 20 ~ 30 years without treatment). Recent studies have indicated that infection with *C. sinensis* or *Opisthorchis viverrini* (another kind of liver fluke) can potently induce dysregulation or alteration of intestinal microbiota in patients or mice, showing a significant reduction of *Bacteroides* and anti-inflammatory *Bifidobacterium*, as well as increase of probiotic *Lactobacillus* [16–18]. Given that *C. sinensis* infection can potently alter the intestinal microbiome, we hypothesized that *C. sinensis* infection may re-balance dysbiosis of the intestinal microbiota in DSS-induced colitis, which may increase SBAs and activate SBAs/TGR5 signaling pathway, thus, modulating inflammatory responses and ameliorating the severity of colitis. Our data reveal a new mechanism by which helminth infection protects against inflammatory disorder disease.

## Results

### *C. sinensis* Infection can Ameliorate the Severity of Colitis induced by DSS Administration

**Fig. 1:**
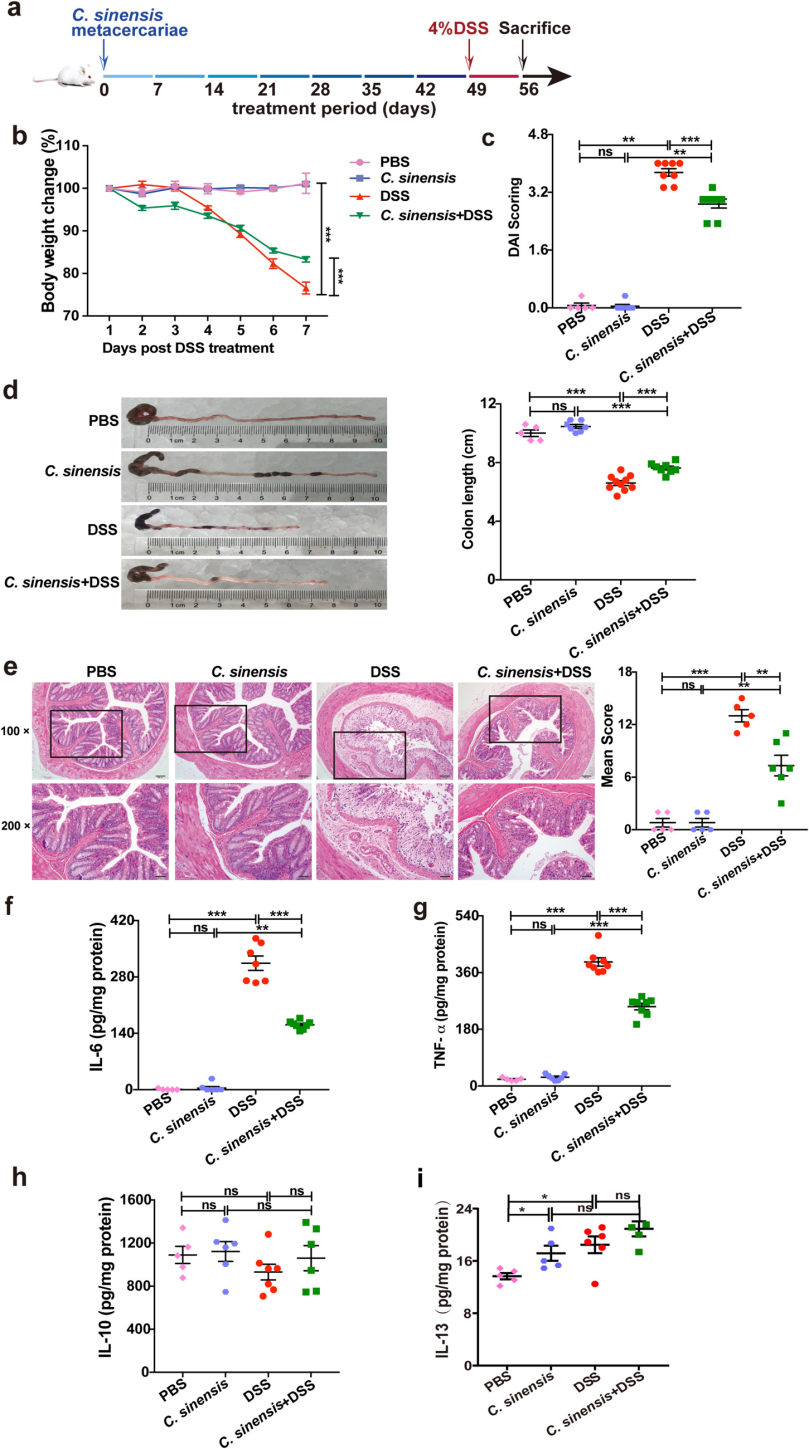
*C. sinensis* infection prevents the mice from DSS-induced experimental colitis. (**a**) The experimental design: BALB/c mice were orally infected with 50 *C. sinensis* metacercariae, followed by administration of 4% DSS water for 7 days on the 49th day of post-infection (*p.i.*), and the mice were sacrificed 56 days post-infection*.* **(b)** The changes in body weight of mice in PBS, *C. sinensis* infection without DSS water-drinking, DSS-water drinking, and DSS + *C. sinensis* infection group. **(c)** DAI scoring. **(d)** The changes in colon length. **(e)** Histopathology score of colitis by H&E staining. **(f ~ i)** The concentration of IL-6 (f), TNF-α (g), IL-10 (h), and IL-13(i) in mice of each group determined by ELISA. Compared with the indicated group, *P < 0.05, ** *P* < 0.01, ***P < 0.001, “ns” means no statistical significance

To investigate the effects of *C. sinensis* infection on the DSS-induced experimental colitis, we employed a well-established *C. sinensis* infection mouse model (sFig 1a-f), and *C. sinensis*-infected mice were administrated with 4% DSS water drinking for 7 days on the 49th day of post-infection (Fig. 1a). The data showed that DSS-administrated mice without infection showed a significant loss of body weight, a reduction of colon length, and increased disease activity index (DAI) and histopathology scores, compared with phosphate-buffered saline (PBS) -drinking mice. Furthermore, as shown in Fig. 1b and 1c, body weight (Fig. 1b, *P* > 0.05) and DAI (Fig. 1c, *P* > 0.05) in the mice infected with *C. sinensis* remained unchanged, compared with the PBS group. Additionally, no significant change in colon length was observed (Fig. 1d, *P* > 0.05), suggesting that *C. sinensis* infection did not affect body weight, DAI, and colon length in BALB/c mice during the experiment. However, *C. sinensis*-infected mice ameliorated these assessments of colitis induced by DSS drinking water (Fig. 1e, *P*< 0.01). We also assessed pro-inflammatory cytokines, such as interleukin-6 (IL-6), tumor necrosis factor-α (TNF-α), and regulatory cytokines interleukin-10 (IL-10), and found that the levels of IL-6 and TNF-α in the colon of *C. sinensis*-infected mice with DSS administration were significantly decreased compared with those in non-infected mice which were also treated with DSS drinking water for 7 days (Fig. 1f and g, *P*< 0.001). Surprisingly, there were no changes in IL-10 or IL-13 in the colon of mice among different groups (Fig. 1h and I). These data demonstrate that *C. sinensis* infection with mice protects against DSS-induced experimental colitis and IL-10 is unlikely to contribute to this amelioration.

### *C. sinensis* Infection Protects from the Loss of Integrity of the Intestinal Barrier in DSS-induced Colitis

**Fig. 2:**
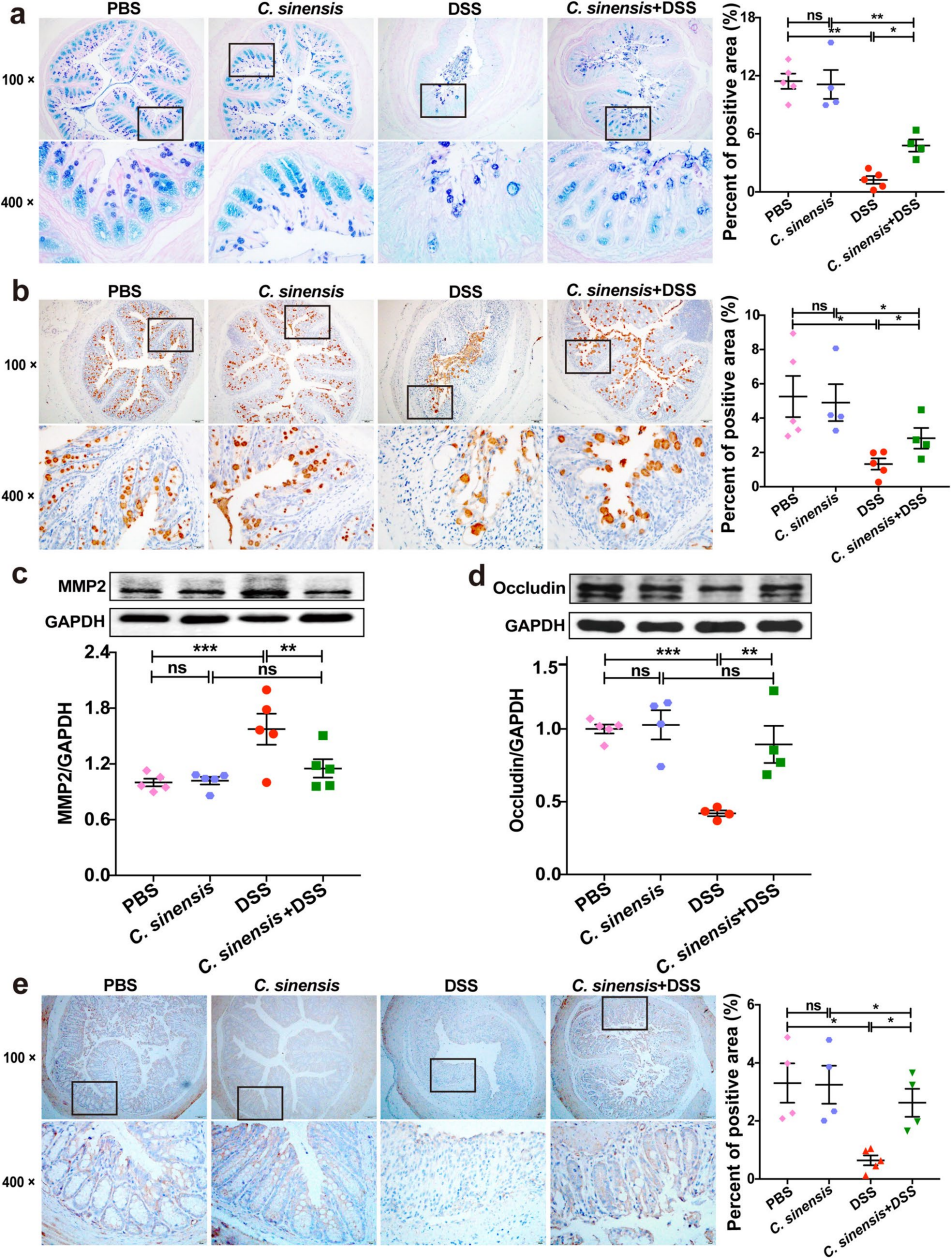
*Clonorchis sinensis* infection ameliorates the injuries of intestinal barriers due to DSS-induced colitis. **(a)** The secretion of mucus in the colon of mice in each group was shown by PAS-Alcian Blue staining. **(b)** The colonic expression of Muc2 of mice in each group was shown by IHC staining, and the percentage of positive area in the total area was analyzed by Image-Pro Plus. **(c)** The expression of MMP2 in the colon of mice in each group was determined by western blot. **(d-e)** the expression of tight junction protein of Occludin (d) and ZO1 (e) in the colon of mice in each group was determined by western blot and IHC, respectively. The percentage of positive area in the total area was analyzed by Image-Pro Plus. n = 4 ~ 5 mice in each group. Compared with the indicated group, *P < 0.05, **P < 0.01, ***P < 0.001, ns means no statistical significance

We also assessed the integrity of the intestinal barrier to investigate the protection of *C. sinensis* infection from colitis. PAS-Alcian Blue staining showed that DSS treatment remarkably reduced the production of mucus in the non-infected mice; however, the secretion of mucus in *C. sinensis*-infected mice was significantly higher than that in non-infected mice (Fig. 2a, *P* < 0.05). Immunohistochemistry (IHC) staining of Mucin 2 (Muc2), which is a key component of the mucous layer and plays a role in the protection of the gut barrier [19] also showed that *C. sinensis* infection in the DSS-induced colitis increased the expression of Muc2 in the cells that are positive by PAS-Alcian Blue in the intestine, compared with non-infected mice with DSS treatment (Fig. 2b, *P* < 0.05). We also found that the level of Matrix Metalloproteinase 2 (MMP2) in the colon of non-infection mice, an enzyme capable of cleaving extracellular matrix, thus destroying the intestinal barrier was augmented due to DSS administration, compared with the PBS group (Fig. 2c, *P* < 0.001); however, in the mice with colitis, *C. sinensis* infection significantly decreased the expression of MMP2 in the colon of mice, compared with non-infected mice (Fig. 2c, *P* < 0.01). Occludin and zonula occludens-1 (ZO1) are two tight junction proteins that are also used to indicate the integrity of the gut barrier. The levels of Occludin and ZO1 in the colon of *C. sinensis*-infected mice were both significantly higher than those in non-infected mice when these mice were subjected to DSS drinking (Fig. 2d and e, *P* < 0.05). Taken together, our data demonstrate that *C. sinensis* infection promotes the intestinal barrier integrity in experimental colitis.

### *C. sinensis* Infection Modulates the Dysbiosis of the Intestinal Microbiota caused by DSS-induced Colitis

**Fig. 3:**
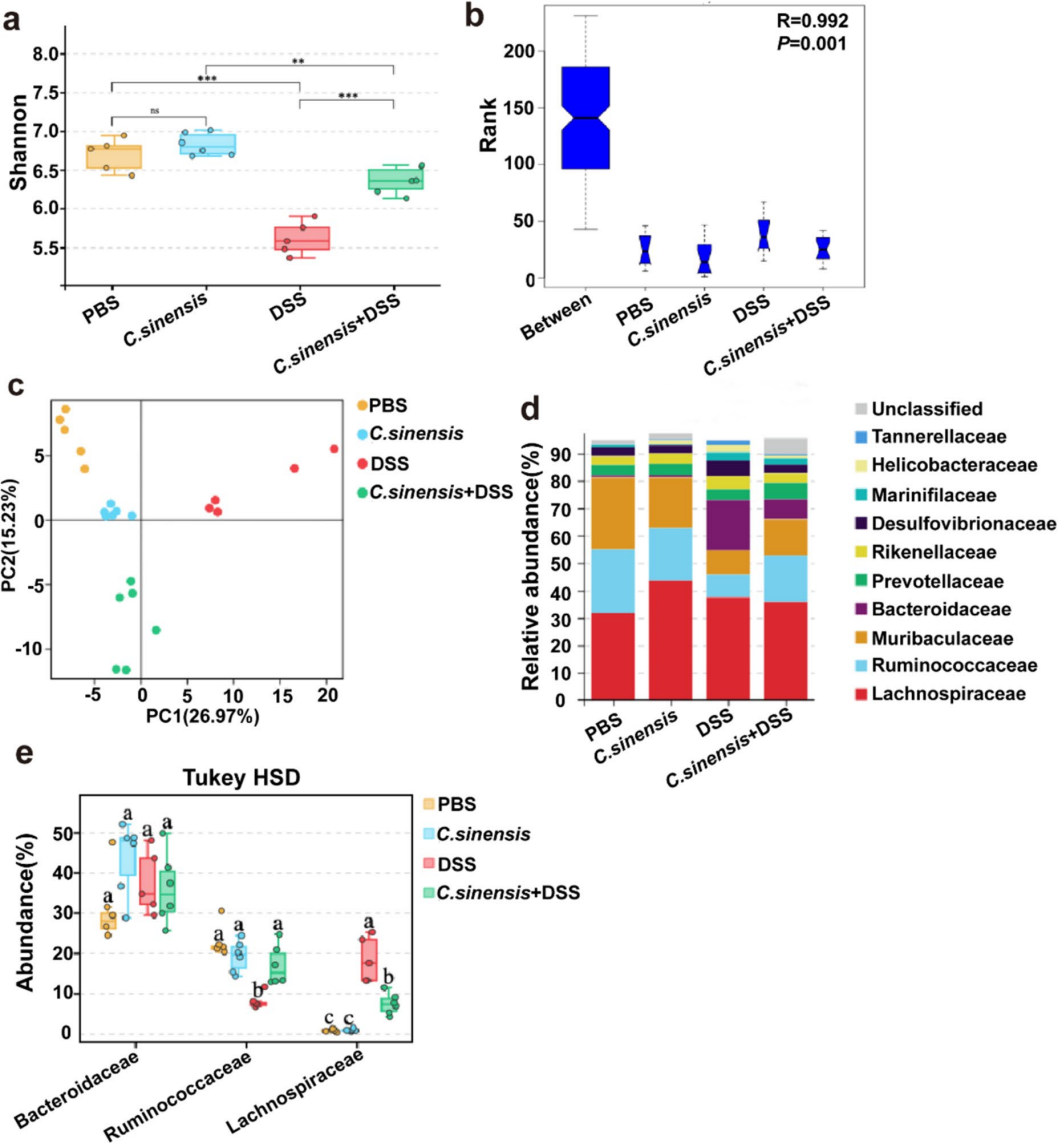
*C. sinensis* rebalanced the gut microbiota in the colitis induced by DSS. The mice were treated as described in Fig. 1a. Upon the mice were sacrificed, the cecal contents of each mouse were collected, and 16S rDNA high-throughput sequencing was used to detect the cecal bacterial diversity and structures. **(a)** Shannon index showed α diversity of gut flora. **(b)** Bray-Anosim test. **(c)** Principal Component Analysis.** (d)** The Structure and composition of the gut microbiome in the mice of each group. (**e**) The abundance of *Rumsfiguinococcaceae*, *Bacteroidaceae*, and *Lachnospiracae* in the mice of each group. n = 5 ~ 6 mice in each group. Compared with the indicated group, * *P* < 0.05, **P < 0.01, ***P < 0.001

To investigate whether *C. sinensis* infection can re-balance DSS-induced dysbiosis of intestinal microbiota or not, we employed a bacterial 16S rDNA high-throughput sequencing assay to analyze bacterial communities in the colon of mice in each group. Sequencing data and qualified sequencing saturation found that there was no significant difference in α diversity of microbiota in the colon of *C. sinensis* infection without DSS administration and PBS group (sFig. 2a). However, it showed a significant decrease in microbial α-diversity in the DSS-treated mice, compared to the PBS control mice; however, microbial α-diversity was significantly higher in the *C. sinensis*-infected mice with DSS compared to the worm-infected mice with DSS (Fig. 3a and sFig. 2b, *P* < 0.001). By the Bray-Anosim test, the R-value is 0.992, and the P value is 0.001, which indicates that the β-diversity is significantly different among the four groups (Fig. 3b). In detail, there was more similarity of β-diversity among the mice of PBS, *C. sinensis* and *C. sinensis* with DSS treatment, but a significant difference with the DSS group (Fig. 3c P = 0.001). It also showed that Differential abundance analysis revealed that the microbial structure of DSS-treated mice was severely disrupted, shifting from the *Bacillota* to the *Bacteroidota* (sFig. 2c), whereas *C. sinensis* infection prevented the disruption of the microbial structure in the DSS group (sFig 2d); Furthermore, there was a decrease in the *Ruminococcaceae* and an increase in the *Bacteroidaceae* in the mice of DSS group compared to the PBS group, while the decrease in the *Ruminococcaceae* and increase in the *Bacteroidaceae* in the mice of *C. sinensis* with DSS administration were significantly reversed (Fig. 3d and e). Taken together, these data demonstrate that *C. sinensis* infection can improve α/β-diversity of intestinal microbiota and rebalance the disrupted microbial structure in DSS-induced colitis.

### *C. sinensis* Infection Induces a High Level of SBAs and Increases the Expression of TGR5

**Fig. 4:**
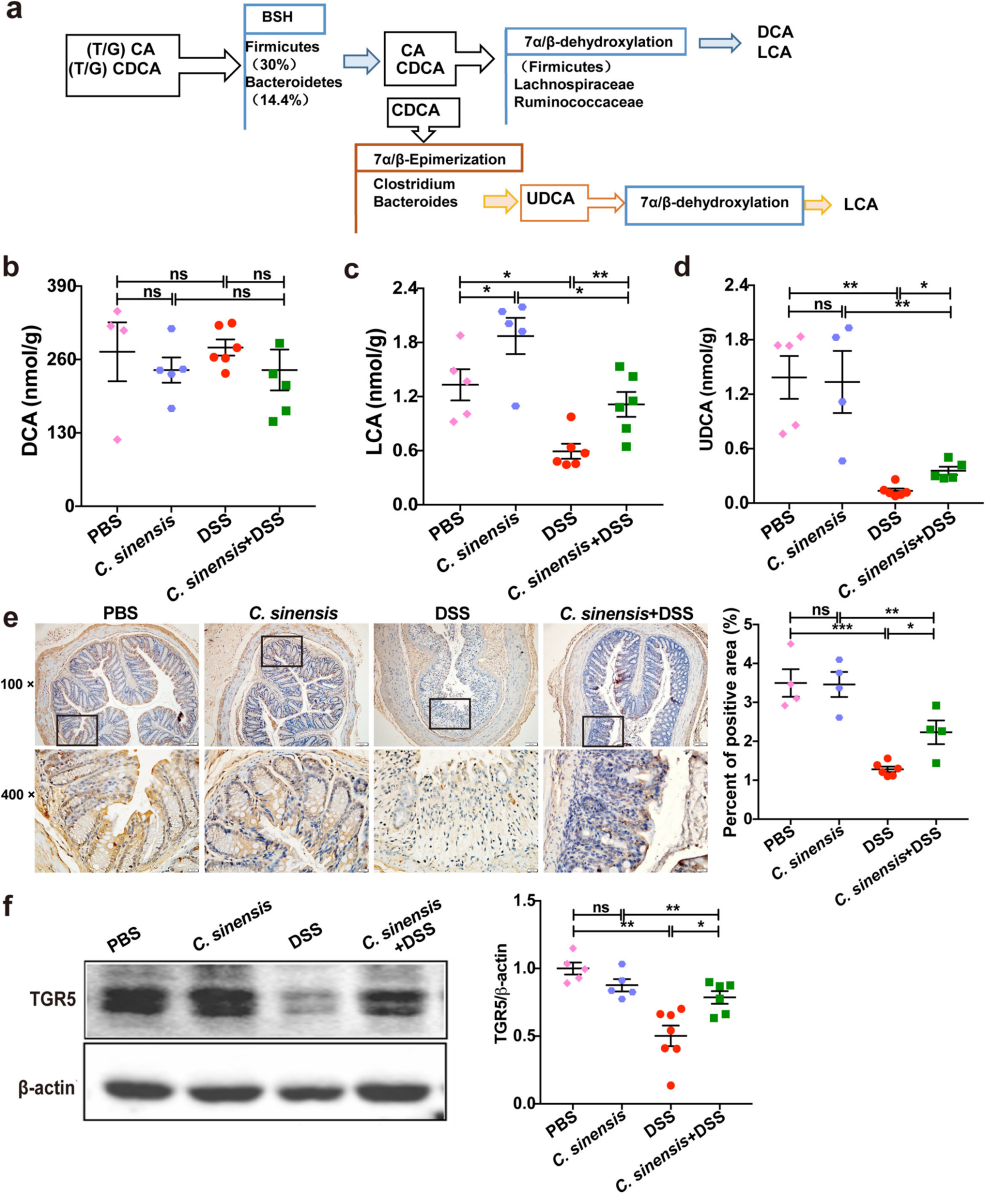
*C. sinensis* infection induced the restoration of gut microbiota-derived SBAs. **(a)** The schematic diagram shows that LCA, DCA, and UDCA are synthesized from PBAs by the bacteria of *Lachnospiraceae*, *Bacteroides,* and *Lachnospiraceae*. **(b ~ d)** The concentration of DCA (b), LCA (c), and UDCA (d) in the cecum of different groups of mice was determined by LC/MS. **(e)** The expression of TGR5 in the colon of different groups of mice was determined by IHC, and the percentage of positive area in the total area was analyzed by Image-Pro Plus. **(f)** TGR5 in the colon was detected by western blot. n = 4 ~ 7 mice in each group. Compared with the indicated group, *P < 0.05, **P < 0.01, ***P < 0.001, “ns” means no statistical significance

As *Ruminococcus* and *Lachnospiraceae* are key microbes that can convert PBAs to SBAs (Fig. 4a) [9, 20], we next screened bile acids (including PBAs and SBAs) using LC–MS. The data showed that there was no significant difference in PBAs including cholic acid (CA) and chenodeoxycholic acid (CDCA), glycocholic acid (GCA), glycochenodeoxycholic acid (GCDCA), glycodeoxycholic acid (GDCA), glycolithocholic acid (GLCA), taurocholic acid (TCA), taurochenodeoxycholic acid (TCDCA), taurodeoxycholic acid (TDCA), taurolithocholic acid (TLCA), glycoursodeoxycholic acid (GUDCA) and tauroursodeoxycholic acid (TUDCA) (sFig. 3a-l), as well as secondary bile acid-deoxycholic acid (DCA, Fig. 4b) in the mice among these groups. However, we found that some secondary bile acids such as LCA and ursodeoxycholic acid (UDCA) were significantly decreased in the colitis of mice without infection, compared with PBS control, but *C. sinensis* infection augmented the levels of LCA and UDCA in the colon, which were decreased by DSS-induced colitis (Fig. 4c and d, *P* < 0.05).

Since SBAs (such as LCA) can act as physiologic ligands for TGR5 (also known as GPBAR1) to regulate intestinal immunity, and LCA has the strongest binding activities with TGR5 among these SBAs[8], we detected the expression of TGR5 in the colon of mice. IHCs and western blot both showed that the expression of TGR5 in the mice without worm infection was significantly decreased after DSS treatment, compared with the normal control group (*P* < 0.01); however, the expression of TGR5 in the *C. sinensis*-infected mice with colitis was significantly elevated, compared with the DSS-administrated mice without worm-infection (Fig. 4e and f, *P* < 0.05). These data suggest that SBAs LCA/UDCA and its bile acid receptor TGR5 may be involved in the worm-induced amelioration of DSS-induced colitis.

### The Intestinal Microbiota is Critical to *C. sinensis*-induced Protection from Colitis

**Fig. 5:**
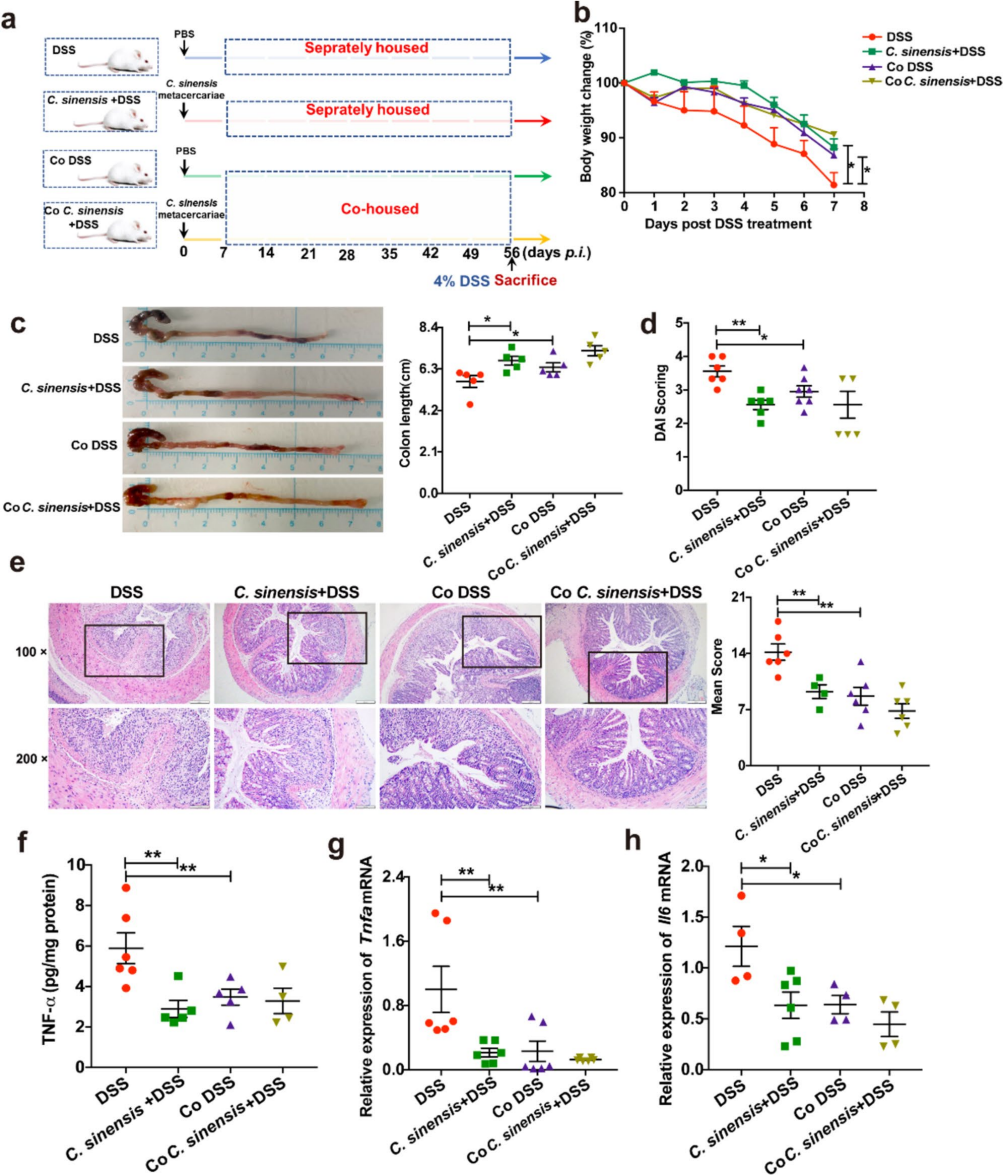
Non-infected mice co-housing with *C. sinensis* infected mice showed amelioration of colitis. **(a)** The mice were intragastrically administrated by *C. sinensis* (*C. sinensis*-infected mice) or PBS (Non-infected mice). All mice were exposed to 4% DSS in their drinking water for 7 days, starting on the 49th day post-infection. Non-infected mice and infected mice separately housed were named the DSS group and *C. sinensis* + DSS group, respectively. Non-infected mice co-housed with *Clonorchis sinensis*-infected mice from the 7th day of infection until the end of the infection were designated as the Co DSS group and Co *C. sinensis* + DSS group, respectively. **(b)** The loss of body weight. **(c)** The length of the colon. **(d)** The DAI. **(e)** the histopathology score of colitis by H&E staining. **(f)** The concentration of TNF-α. **(g&h)** the levels of *Tnfa* and *Il6* mRNAs in the colon of mice. n = 4 ~ 7 mice in each group. Compared with the indicated group, * *P* < 0.05, **P < 0.01, ***P < 0.001

To investigate the role of intestinal microbiota in the *C. sinensis*-induced suppression of colitis, we used a co-housing approach that allowed microbiota to be transferred from *C. sinensis*-infected mice to non-infected mice in the condition of DSS-induced colitis (Fig. 5a) [21, 22]. Our data showed that non-infected mice co-housing with *C. sinensis*-infected mice (Co DSS group) showed reduced weight loss (Fig. 5b, *P* < 0.05), increased colonic length (Fig. 5c, *P* < 0.05), decreased DAI (Fig. 5d, *P* < 0.05), compared to the non-infected mice without co-housing in the DSS-induced colitis (DSS group). In addition, we also found that the pro-inflammatory cytokines TNF-α and IL-6 were abated in the colon of mice from the Co DSS group, compared with the DSS group (Fig. 5f ~ h, *P* < 0.05). However, there is still no change in IL-10 at protein or mRNA levels in the colon of mice among these groups (sFig. 4a and b). These results indicated that DSS-induced colitis could be ameliorated by *C. sinensis* in a gut microbiota-dependent manner.

**Fig. 6:**
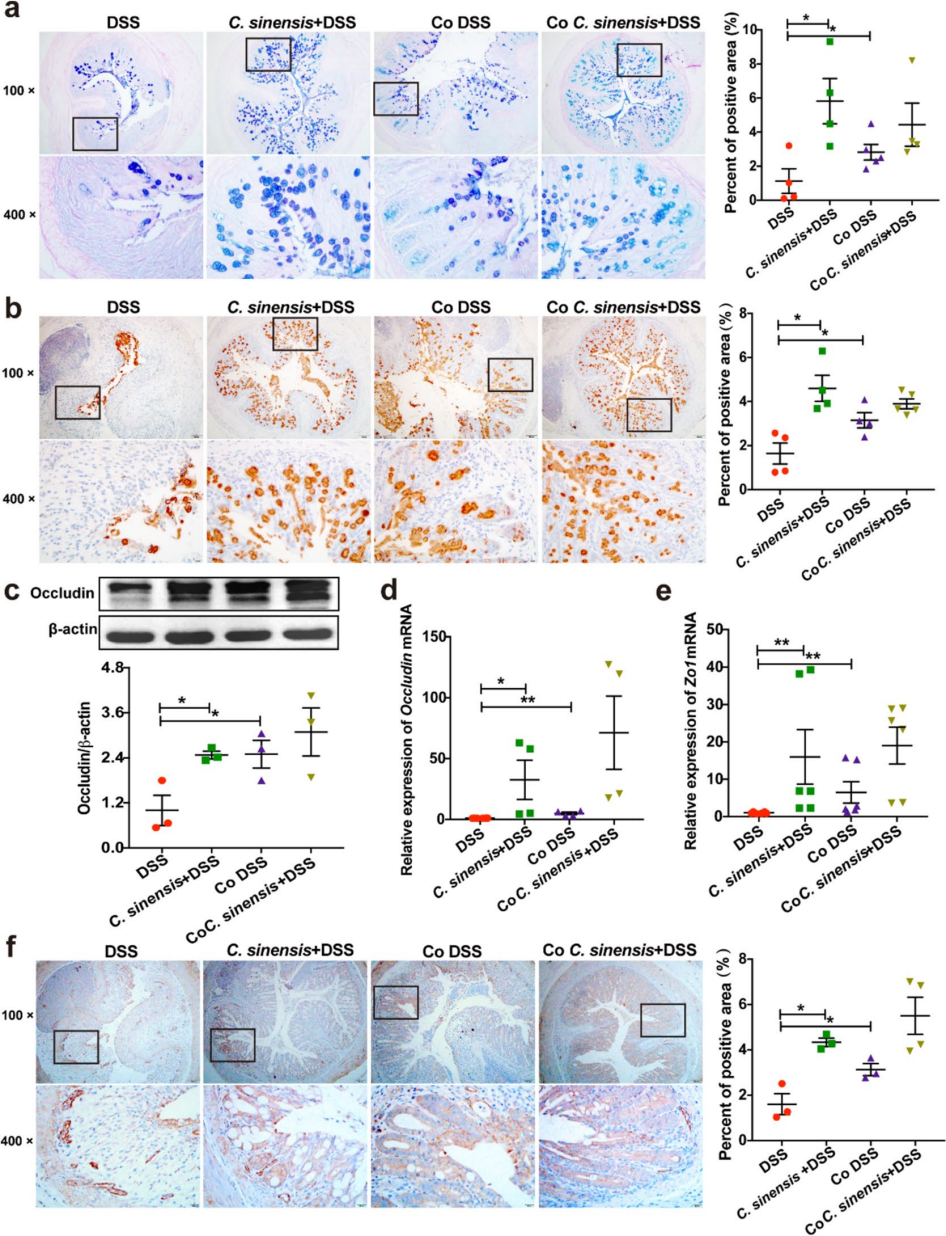
Intestinal injuries are meliorated in non-infected mice after co-housed with *C. sinensis*-infected mice in DSS-induced colitis. **(a)** The secretion of mucus in the colon of mice in the indicated group was shown by PAS-Alcian Blue staining. **(b)** The colonic expression of Muc2 of mice in the indicated group was shown by IHC staining, and the percentage of positive area in the total area was analyzed by Image-Pro Plus. **(c)** The expression of Occludin 2 in the colon of mice in indicated mice was determined by western blot. **(d&e)** The relative expression of *Occludin* and *Zo1* mRNA transcripts in the colon of indicated mice was determined by qPCR. **(f)** The colonic expression of ZO1 of mice in the indicated group was shown by IHC staining, and the percentage of positive area in the total area was analyzed by Image-Pro Plus. n = 3 ~ 6 mice in each group. Compared with the indicated group, * *P* < 0.05, **P < 0.01, ***P < 0.001

We further investigated the roles of gut microbiota in the *C. sinensis*-induced protection from intestinal injuries due to DSS-induced colitis using the co-housing assay. We found that non-infected mice co-housing with *C. sinensis*-infected mice also showed increased mucus production and the expression of Muc2 by PAS-Alcian Blue staining (Fig. 6a, *P* < 0.05) and IHC staining (Fig. 6b, *P* < 0.05), respectively, suggesting a better function of intestinal secretion in non-infected mice co-housing with infected mice. Furthermore, the expression of tight junction proteins such as Occludin and ZO1 was also increased in the colon of non-infected mice co-housing with *C. sinensis*-infected mice, compared with non-infected mice without co-housing (Fig. 6c and 6d for Occludin determined by western blot and qPCR, respectively,* P* < 0.05; Fig. 6e and f for *Zo1* determined by qPCR and IHC staining, respectively, *P* < 0.05). These data suggest that gut microbiota orchestrates *C. sinensis*-evoked suppression of DSS-induced colitis and prevents intestinal barrier damage caused by DSS-induced colitis.

### Intestinal Microbiota-Derived LCA and UDCA are Associated with *C. sinensis*-Induced Suppression of Colitis

**Fig. 7:**
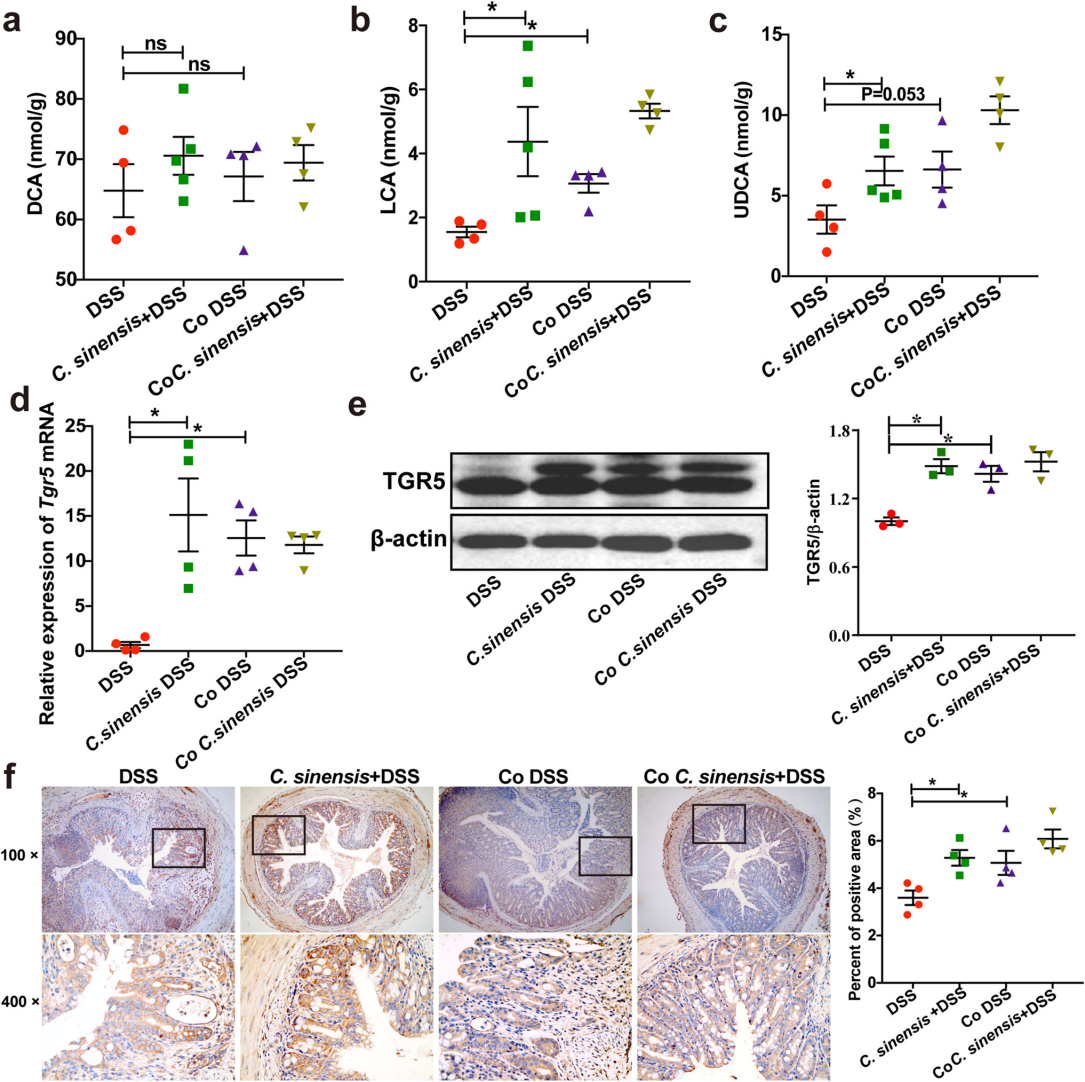
Gut microbiota-derived SBAs and its bile acid receptor TGR5 are regained in non-infected mice after being co-housed with *C. sinensis*-infected mice in DSS-induced colitis. **(a)** The concentration of DCA, **(b)** LCA, and **(c)** UDCA in the cecum of different groups of mice was determined by LC/MS. **(d ~ f)** The expression of TGR5 in the colon of different groups of mice was determined by qPCR (d), western blot (e), and IHC staining, and the percentage of positive area in the total area was analyzed by Image-Pro Plus (f). Compared with the indicated group, *P < 0.05, **P < 0.01, ***P < 0.001

We further detected SBAs-DCA, LCA, and UDCA in the colon of mice with or without co-housing in the condition of colitis and found that the concentration of DCA was not changed among these groups while the levels of LCA and UDCA were significantly increased in the non-infected mice co-housing with infected mice, compared with non-infected mice without co-housing (Fig. 7a ~ c, *P* < 0.05). Furthermore, the expression of LCA and UDCA receptor TGR5 was also augmented in the non-infected mice co-housing with C. sinensis-infected mice, as demonstrated by qPCR (Fig. 7d, *P* < 0.05), western blot (Fig. 7e, *P* < 0.05) and IHC staining (Fig. 7f, *P*< 0.05). These data suggest that bacterial transfer from *C. sinensis*-infected mice to non-infected mice due to co-housing induces high levels of BAs, which may contribute to *C. sinensis*-evoked suppression of DSS-induced colitis.

### The Protective effects of *C. sinensis* on Experiment Colitis are Dependent on Microbiota-Mediated SBAs

**Fig. 8:**
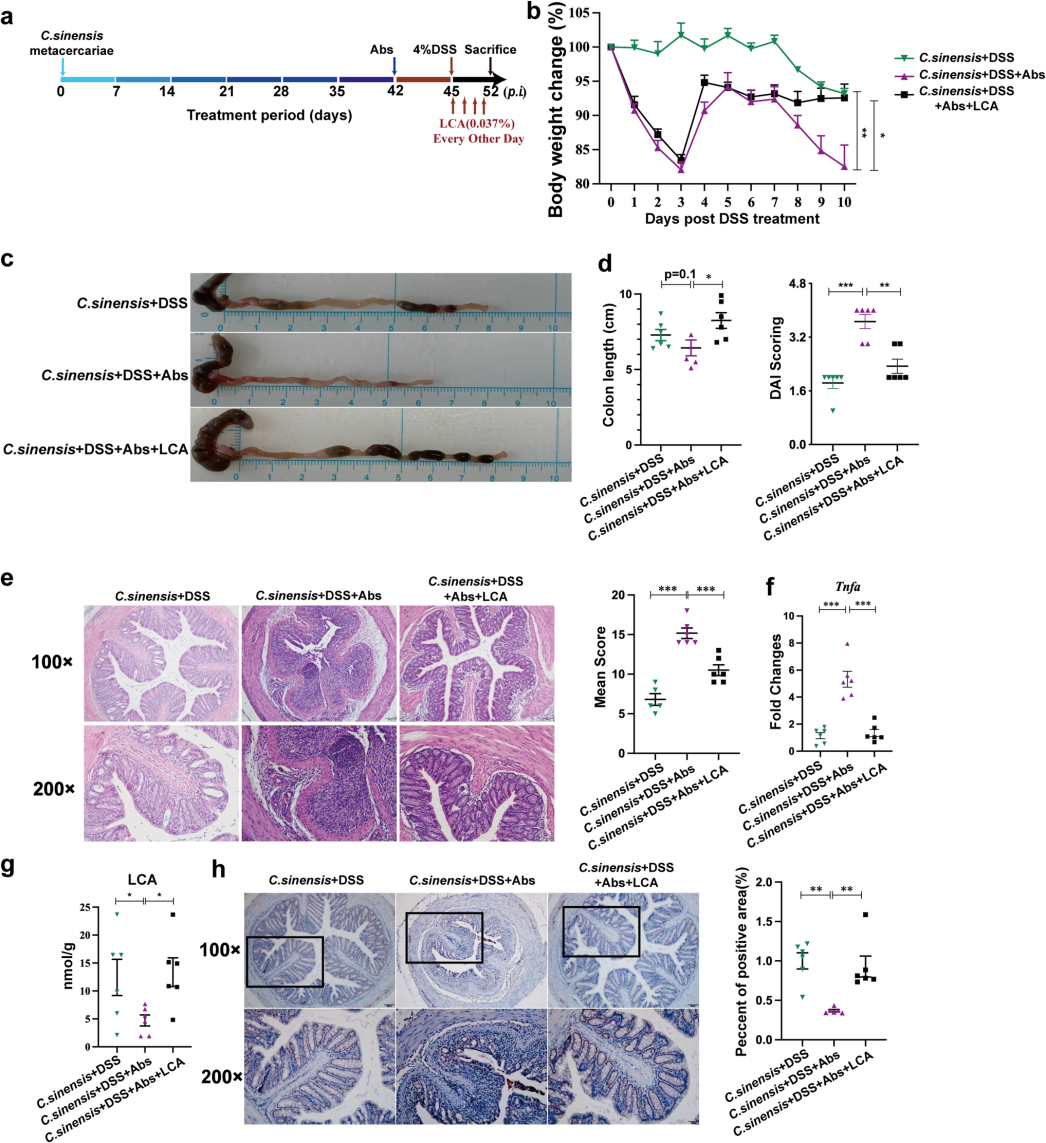
Gut microbiota-mediated LCA is required in the *C. sinensis*-evoked protection of experimental colitis.** (a)** The experimental design: the *C. sinensis* -infected mice were treated with a broad-spectrum cocktail of antibiotics (Abs) and adding LCA (0.037%), and DSS-administrated for experimental colitis on the indicated days of post-infection (*p.i.*). **(b)** The loss of body weight, **(c)** The length of the colon, **(d)** The DAI, **(e)** the histopathology score of colitis by H&E staining. **(f)** The levels of *Tnfa* in the colon of mice. **(g)** The concentration of LCA. **(h)** The percentage of TGR5 positive areas in the colon in each group of mice were evaluated by IHC staining and the percentage of positive area in the total area was analyzed by Image-Pro Plus. n = 4 ~ 8 mice in each group. Compared with the indicated group, * *P* < 0.05, **P < 0.01, ***P < 0.001

To further demonstrate the roles of microbiota-mediated SBAs in *C. sinensis -*induced protection of colitis, we applied a broad-spectrum cocktail of antibiotics (Abs) to delete gut microbiota and subsequently supplied with SBA (LCA) to *C. sinensis* + DSS mice on indicated days (Fig. 8a). The data showed that, compared with *C. sinensis* + DSS mice without antibiotics treatment, mice with antibiotics treatment had more severe weight loss (Fig. 8b, *P* < 0.01), shorter length of colon (Fig. 8c, *P*= 0.1), higher DAI scores (Fig. 8d, *P* < 0.001), more severe pathological damage (Fig. 8e, shown by H&E staining and semiquantitative, *P* < 0.001), and significantly increased pro-inflammatory cytokine *Tnfa* (Fig. 8f, *P*< 0.001). However, additional supplementation with LCA (0.037%) to the *C. sinensis* + DSS mice with gut microbiota deletion, the colitis of these mice as shown by the above indicators was significantly reversed and showed amelioration of colitis (Fig. 8b ~ f, *P* < 0.05).

We further detected the concentration of LCA in the *C. sinensis* + DSS mice with or without gut microbiota deletion and found that the concentrations of LCA in antibiotics-treated mice were significantly decreased, compared with those *C. sinensis* + DSS mice without antibiotics treatment, suggesting that endogenous LCA can be blocked by antibiotics and the gut microbiota is the main producer of LCA (Fig. 8g, *P* < 0.05). Supplementary exogenous LCA (0.037%) can increase the concentration of LCA in the gut (Fig. 8g, *P* < 0.05). Furthermore, we also detected the expression of TGR5, it was found that the level of TGR5 in the *C. sinensis* + DSS mice with gut microbiota deletion was significantly decreased compared with those mice without gut microbiota deletion (Fig. 8h, *P* < 0.01); however, when Supplementary exogenous LCA was administrated to the *C. sinensis* + DSS mice with gut microbiota deletion, the level of TGR5 was significantly augmented, compared with those mice without LCA supplementation (Fig. 8h, *P* < 0.001). Taken together, these data demonstrate that gut microbiota-mediated LCA is critical to *C. sinensis*-evoked protection from colitis.

## Discussion

**Fig. 9:**
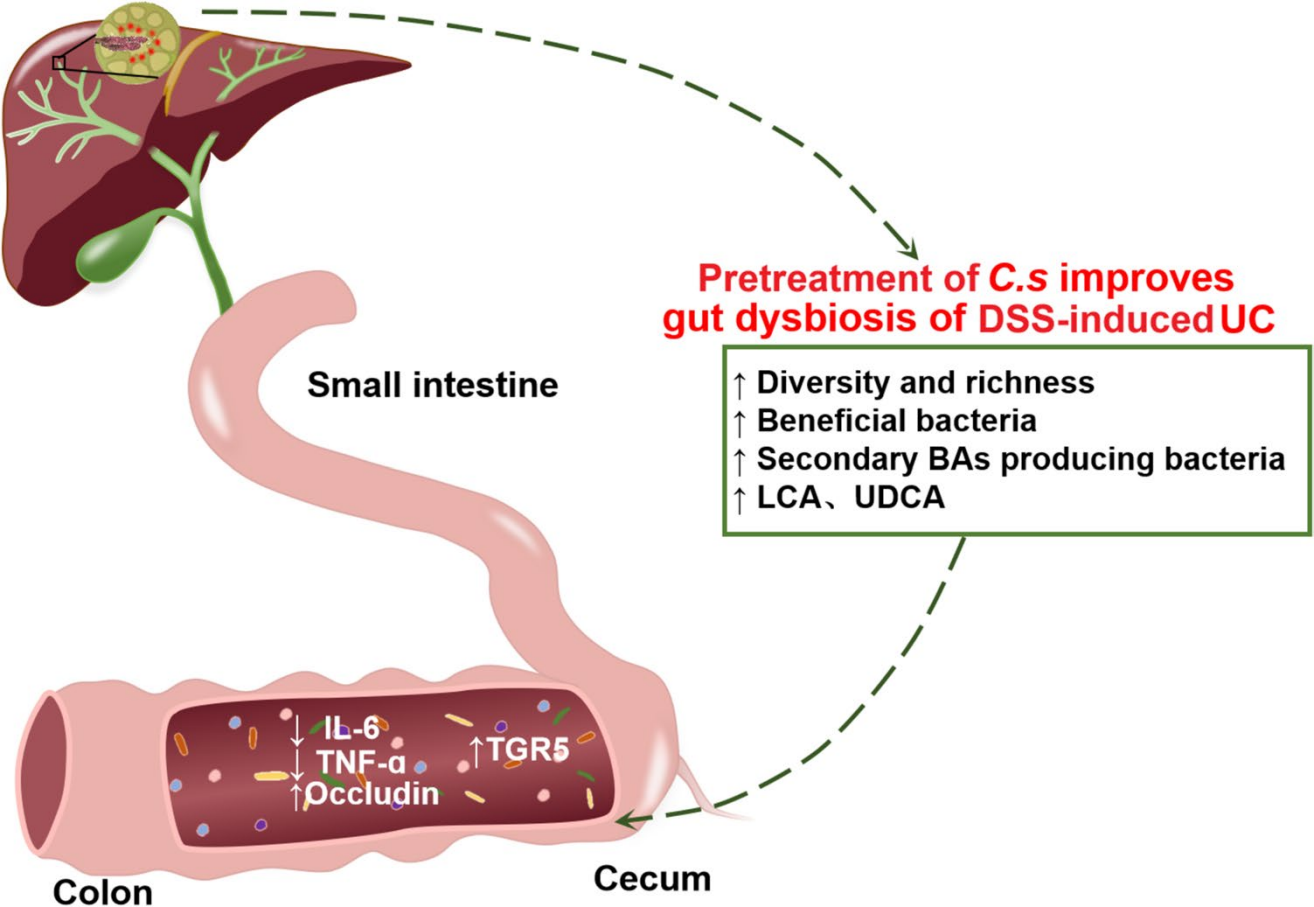
*Clonorchis sinensis* evokes protection of DSS-induced experimental ulcerative colitis in mice dependent on intestinal microbiota-mediated bile acids

The hygiene hypothesis has been proposed for more than 30 years, but the mechanism underlying helminth protection from these inflammatory diseases is largely unknown. In this study, we found that liver fluke-*C. sinensis* infection alleviated experimental colitis in mice by the modulation of the intestinal microbiota and its metabolite SBAs. The co-housing assay with *C. sinensis*-infected mice demonstrated that non-infected mice administrated DSS showed an increase of SBAs/TGR5 and a reduction in the severity of colitis, suggesting the critical role of intestinal microbiota (Fig. 9). Furthermore, Microbiota deletion with supplied SBAs assays demonstrated that the meliorative colitis caused by *C. sinensis* infection is dependent on gut microbiota-mediated SBAs. Therefore, we highlight the critical roles of gut microbiota and its metabolite secondary bile acids in the helminth-evoked protection from colitis, which provides evidence to uncover a new mechanism by which helminth infection protects from such inflammatory disorders.

Several mechanisms contribute to the helminth-induced protection from colitis, including suppression of type 1 immunity and triggering immunoregulatory cells and mediators[12, 23]. Of these, the regulatory cytokine IL-10 plays a very important role in intestinal homeostasis and suppression of colitis [15, 24, 25]. Increasing experimental data demonstrate that exposure to a certain helminth such as *T. spiralis*, *Hymenolepis diminuta,* and *Schistosoma mansoni* can potently prevent or attenuate colitis by increasing IL-10 production[26]. Our previous study also showed that *Clonorchis sinensis* Molecular chaperones HscB (CsHscB) derived from *C. sinensis* can attenuate DSS-induced acute and chronic colitis by inducing a high level of IL-10 [27, 28]. Similar to these data, a recent study also showed that crude antigen (CsCA) and cysteine protease (CsCP) from *C. sinensis* alleviated acute colitis [29]. Furthermore, it seems that *C. sinensis* infection provided some degree of relief from acute colitis after 35 days post-infection[29]. However, in contrast to the studies above, in our present study, we found that *C. sinensis* infection (49-day post-infection) suppressed colonic pro-inflammatory cytokines such as IL-6 and TNF-α without increaseing IL-10 production, suggesting that *C. sinensis*-induced suppression of colitis is IL-10-independent and another potential mechanism is involved.

The gut microbiota is essential in maintaining the physiological function and homeostasis of the gut [30–32]. Recent studies have demonstrated the significance of the intestinal microbiome in colitis and its regulation by helminth infections[33, 34]. Dysbiosis of the gut microbiome is one of the main characteristics of UC patients. However, the role of gut microbiome in the helminth-induced suppression of colitis has been rarely reported[15]. The loss of gut microbiota α/β diversity, decreased Bacillota, and increased *Lachnospiraceae* were found in the UC patients[6], which was in line with our observation that shifting from the *Bacillota* to the *Bacteroidota* in DSS-induced experimental colitis. However, the dysbiosis of the gut microbiome was prevented in the DSS group with *C. sinensis* infection, although the mechanisms by which *C. sinensis* infection altered microbial structure should be investigated further.

In the colon and cecum, nearly all primary bile acids (PBAs) can be transformed into SBAs (such as LCA, UDCA, and DCA) by 7α-dehydroxylation of bile acid-transforming gut microbiota, including some bacteria of *Ruminococcus* family and *Lachnospiraceae* [9, 20]. It has been reported that the lowering of SBAs (LCA and DCA) was associated with intestinal inflammation in UC patients and supplementation of these SBAs reduced the intestinal inflammation[8, 35]. UDCA can be converted into LCA by the microbes of the family *Trichospiraceae* and *Ruminococcus* [9]. In our present study, we found that compared to the DSS group, the levels of UDCA and LCA (but not DCA) were increased, and *Ruminococcus* became predominant in the cecum of DSS mice with *C. sinensis*. Therefore, it seems that LCA among these SBAs may play a predominant role in the *C. sinensis*-infection-evoked protection of colitis.

LCA is the natural ligand of TGR5 with the highest activity among these BAs [36]. Bile acids can be sensed by farnesoid X receptor (FXR) and TGR5, which mediate signal pathways to regulate the expression of genes involved in the metabolism of BA, lipids, and carbohydrates and energy expenditure and inflammation [37]. We found that TGR5 was significantly increased in *C. sinensis*-infected mice with colitis, suggesting that microbial sourced-LC-mediated TGR5 axis may be responsible for the *C. sinensis*-infection evoked protection of colitis, but other LCA-activated bile acid receptors such as FXR, vitamin D receptor (VDR), and sphingosine-1-phosphate receptor 2 (S1PR2), which may also be involved in the process, were not ruled out.

Although our present study demonstrated that live worm infection can protect against colitis, the rational approach to utilizing *C. sinensis* as a treatment for UC is to determine the desirable protective mechanism, such as worm-orchestrated gut microbiota and its metabolite SBAs [34]. Therefore, we should be cautious in interpreting our data and we do not advocate infection of patients with *C. sinensis* worms, because *C. sinensis* infection can also cause severe biliary injuries. Further studies are required to elucidate the exact mechanisms by which these microbial-derived BAs are protected against colitis in the context of helminth infection.

## Materials and Methods

### Ethics Statement

Animal care and all experiments performed in this study were strictly conformed to the guidelines of the National Laboratory Animal Center. The main procedures and protocol were reviewed and approved by the Animal Care and Use Committee of Xuzhou Medical University License (202011A119).

### Preparation of C. sinensis Metacercaria

The metacercariae of *C. sinensis* were obtained as described in our previous study [38]. Briefly, the intermediate host fish *Pseudorasbora Parva* was purchased from Guangxi Province of China and *C. sinensis* metacercariae inside the meat of fish were screened and checked. The meat of positive fish was digested overnight with pepsin-HCl (0.6%) artificial gastric juice. The digestion of fish meat was filtered through a series of sieves to remove undesired particles such as fish bone and fish scale. The metacercariae were identified and harvested using a microscope and stored in the refrigerator at 4 °C in phosphate-buffered solution (PBS).

### Experimental Animals

BALB/c mice (female, 5 ~ 7 weeks, weighing 18 ~ 22 g) purchased from the Beijing Vital River Laboratory Animal Technology Co., Ltd. were used for the study. All the mice were housed and bred under specific pathogen-free conditions (temperature 22 °C, 12 h light/dark cycle) at the animal center of Xuzhou Medical University.

### *C. sinensis* Infection and DSS Drinking Water

BALB/c mice were randomly divided into 4 groups (n = 5 ~ 10 in each group): PBS group, *C. sinensis* group, DSS group, and *C. sinensis* + DSS group. On the 0 days of post-infection (*p.i.*), every mouse in the group of *C. sinensis* and *C. sinensis* + DSS was intragastrically administered with 50 metacercariae, and the other two groups were given the same volume of PBS. The mice of the DSS group and *C. sinensis* + DSS group were given 4% Dextran Sodium Sulfate (DSS, mol. wt. 36,000 ~ 50,000, MP Biochemicals, Santa Ana, CA) free drinking water for 7 days which started from the 49-day *p.i.*, and the other two groups were given PBS drinking water, and the daily changes in body weight, stool traits and fecal occult blood of each mouse were recorded for disease activity index (DAI)[39]. All mice were sacrificed by cervical dislocation immediately after exsanguination on 56-day *p.i.*, and the colon tissues and cecal contents were collected for further study. The timeline of this animal experiment is shown in Fig. 1A.

### Co-Housing Study

BALB/c mice were randomly divided into 4 groups (n = 5 ~ 7 in each group): DSS group, *C. sinensis* + DSS group, Co DSS group, and Co *C. sinensis* + DSS group. All mice were exposed to 4% DSS in their drinking water. Non-infected mice and infected mice separately housed were named the DSS group and *C. sinensis* + DSS group, respectively. Non-infected mice co-housed with *Clonorchis sinensis*-infected mice from the eighth day of infection until the end of the infection were designated as the Co DSS group and Co *C. sinensis* + DSS group, respectively. Detaily, on the 0 day of post-infection (*p.i.*), every mouse in the group of *C. sinensis* + DSS and Co *C. sinensis* + DSS was intragastrically administered with 50 metacercariae, and the other two groups were given the same volume of PBS. Starting from the 7-day *p.i.*, mice in the group of Co *C. sinensis* + DSS and Co DSS were caged together for cohousing study. All groups of mice were administered 4% DSS drinking water for 7 days starting from the 49-day *p.i.*. The DAI score of each mouse was recorded. All mice were sacrificed on 56-day *p.i.*, and the colon tissues and cecal contents were collected for further study.

### Microbiota Deletion and LCA Treatment

Mice were orally infected with *C. sinensis* as described above. For deletion of gut microbiota, the infected mice were provided with water containing a broad-spectrum cocktail of antibiotics (Ampicillin sodium, 1 g/L, A100339-0005, Sangon Biotech, Shanghai; Neomycin sulfate, 1 g/L, N6386-5G, Sigma; Metronidazole, 0.75 g/L, A600633-0025, Sangon Biotech, Shanghai; Vancomycin, 0.35 g/L, A600983-0001, Sangon Biotech, Shanghai) from the 42-day *p.i.* for 3 days. On the day of 45-day *p.i.*, the water containing antibiotics was replaced with 4% DSS water for 7 days to establish the experimental ulcerative colitis. For LCA treatment, the mice were treated with water containing 0.03% LCA every two other days (a total of 4 times) after DSS treatment[8]. All the mice were sacrificed on 52-day *p.i.*

### Hematoxylin and Eosin (H&E) Staining

For histological analysis, distal colon tissue (about 1 cm) was excised and immersed in 4% paraformaldehyde for 48 h. The tissue was then embedded in paraffin, sliced to a thickness of 4 μm, and stained with hematoxylin (G1005-1, Servicebio, Wuhan, China) or eosin (G1005-2, Servicebio, Wuhan, China) according to the manufacturer’s instructions. The pathological changes in stained sections were analyzed by the Nancy histological index system using a microscope (Olympus, Japan)[40].

### Alcian Blue Periodic Acid Schiff (PAS-Alcian Blue) Staining

PAS-Alcian Blue staining of colon tissue from the mice was performed using 4-μm serial thick sections. The slides were stained according to the manufacturer’s instructions for the PAS-Alcian Blue stain kit (G1285; Solarbio; Beijing; China). The entire tissue (1 to 2 non—overlapping images under low—power fields) was photographed (× 100 magnifications, Olympus, Japan). The percentage of positive area was analyzed using Image J software (NIH, Bethesda, US).

## ELISA

The concentration of cytokines in the colon was detected using Enzyme-linked Immunosorbent Assay (ELISA). In each group, colon homogenate from each mouse was immediately collected to evaluate the concentrations of IL-6, IL-10, IL-13, and TNF-α by a commercial ELISA Kit with 96 well plates (Thermo Scientific, CA, USA). All procedures were performed following the instructions provided by the ELISA kit. Concentrations of cytokines in the colon were calculated using standard curves as a reference.

### Western Blot

Colon tissue homogenates from all mice were collected in microtubes and centrifuged for 15 min at 12 000 rpm at 4℃. The protein concentration was determined using a BCA kit (P0010, Beyotime Biotechnology, Shanghai, China). The proteins were then separated on a 10% SDS polyacrylamide gel electrophoresis and transferred onto 0.45 μm polyvinylidene fluoride nitrocellulose membranes (1,620,177, Bio-Rad, California, USA). After 60 min of blocking with 5% fat-free milk, the membranes were incubated with matrix metallopeptidase 2 (MMP2) (1:500; BS72289, Bioworld, Nanjing, China), Occludin (1:500; AP0765, Bioworld, Nanjing, China) and β-actin antibody (1:5 000; AC026, Abclonal, Wuhan, China) at 4℃ overnight. The membranes were washed and incubated with the anti-rabbit secondary antibody (1:2 000; 7074, Cell Signaling Technology, USA) for 1 h. After washing, the blots were visualized using an enhanced chemiluminescence kit (Jiangsu Beyotime Biotechnology Research Institute, China).

### Immunohistochemistry (IHC) Staining

Immunohistological analysis of the colon tissue was performed using 4-μm serial thick sections of embedded tissue from each mouse. Briefly, the colon tissue was deparaffinized, hydrated, and heated in citric acid buffer at 95 °C for 10 min, and then blocked with 5% bovine serum albumin (BSA) for 30 min. The slides were then incubated overnight with primary Anti-GPCR (TGR5) (1:500; ab72608, Abcam, Cambridge, USA), Muc-2 (1:600; ab272692, Abcam, Cambridge, USA), Zo1 (1:100; ab221546, Abcam, Cambridge, USA). After the incubation, the slides were washed with PBS, and DAB (PV-9001, ZSGB-BIO, Beijing, China) as an enzyme substrate was added. The entire tissue (1 to 2 non—overlapping images under low—power fields) was photographed (× 100 magnifications, Olympus, Japan). The signal intensity was quantified using Image J software (NIH, Bethesda, US).

### Quantitative Real-Time Fluorescence PCR (qRT-PCR)

Total RNAs were extracted from colon tissues using TRNzol Reagent (DP424, TIANGEN Biotech, Beijing, China) in an RNase-free environment according to the manufacturer's protocol, and RNA was reverse transcribed into cDNA using the PrimeScript™ RT reagent Kit (RR037A, TaKaRa, Tokyo, Japan). qRT-PCR was performed on a Roche 480 detection system (Roche Diagnostics Ltd, Shanghai, China) using TB Green® Premix Ex Taq™ (RR420A, TaKaRa, Tokyo, Japan). The relative mRNA expression levels were normalized to *β-actin* following the 2^−△△Ct^ comparative method. Primers for mouse: *β-actin* F: AACTCCATCATGAAGTGTGA; R: ACTCCTGCTTGCTGA TCCAC; *Tnfa* F: ACGGCATGGATCTCAAAGAC; R: AGATAGCAAATCGGCTGACG; *Il6* F: GTTCTCTGGGAAATCGTGGA; R: GGAAATTGGGGTAGGAAGGA; *Il10* F: GCTCTTACTGACTGGCATGAG; R: CGCAGCTCTAGGAGCATGTG; *Occludin* F: TGAAAGTCCACCTCCTTACAGA; R: CCGGATAAAAAGAGTACGCTGG; *Zo1* F: GCCGCTAAGAGCACAGCAA; R: GCCCTCCTTTTAACACATCAGA; *Tgr5* F: CCTGGAACTCTGTTATCGCTCA; R: GCACTCGTAGACACCTTTGGG.

### Gut Microbiota 16S rDNA Sequencing

Cecal contents from the mice (n = 5 ~ 6) were collected and stored at −80 °C. Total DNAs of the microbial community in cecal contents were extracted using the HiPure Stool DNA Kit (D3141, Magen Biotech, Guangzhou, China), and the quality and concentration were evaluated by NanoDrop spectrophotometry (NanoDrop 2000, Thermo Scientific, Wilmington, USA). The nucleic acid integrity was checked by 2% agarose gel electrophoresis. Subsequently, the V3-V4 variable region of the 16S rDNA gene was purified by AMPure XP Beads (Beckman Agencourt, USA) and quantified on the ABI StepOnePlus Real-Time PCR System (Life Technologies, Foster City, USA). Data was analyzed based on the Illumina platform (Illumina, San Diego, CA, USA). In order to acquire high-quality sequencing data, certain sequences were excluded. Specifically, those with a length less than 150 bp, an average Phred score below 20, ambiguous bases, and mononucleotide repeats exceeding 8 bp were removed. Following chimera detection, the remaining high-quality sequences were grouped into operational taxonomic units (OTUs) at a 97% sequence identity level using UCLUST[41]. To guarantee the dependability and precision of the analysis, OTUs whose abundances were lower than 0.001% of the total sequences in all samples were eliminated[42].

### Bioinformatics

Multiple sequence alignment was conducted using the MUSCLE software (version 3.8.31). A bipartite association network, principal coordinate analysis (PCoA) based on Bray–Curtis distance, and Spearman correlation coefficient were implemented using Cytoscape (version 3.7.1) and R programming language (Version. 3.5.3). To gauge the sequencing depth and species abundance, OTU-level species accumulation graphs were deployed. A range of alpha diversity measures, namely Chao 1 richness, the ACE estimator (Abundance-based Coverage Estimator), the Simpson coefficient, and the Shannon diversity metric, were determined with the aid of QIIME. The Kruskal–Wallis statistical tests were employed to pinpoint notable distinctions in alpha diversity measures among distinct groups. The abundances of taxa at the phylum and genus tiers were scrutinized through the Metastats functionality within Mothur and then graphically represented as violin diagrams. Linear discriminant analysis effect size (LEfSe) was carried out to uncover taxa with differential abundances between groups, adhering to the default parameters, and the threshold logarithmic linear discriminant analysis (LDA) value was established at 2.0[43].

### Bile Acids Detection

For the analysis of bile acids, bile acids based on targeted metabolomics analysis were performed by LC–MS using the multiple reaction monitoring (MRM) mode and quantified based on the respective standard curves. In brief, 10 mg of cecum contents were homogenized in 300 μL of extractant containing 0.1% formic acid (methanol: acetonitrile: ultrapure water = 2: 2: 1) for 3 min (Ultrasound in the ice bath for 5 s, pause for 5 s) and then centrifuged at 15 000 g for 10 min at 4 °C. Supernatants were analyzed for LC–MS at 4 °C in the Xuzhou Maternal and Child Health Hospital Affiliated with Xuzhou Medical University. MS data were processed using the SCIEX OS software and analyzed with Microsoft Excel.

### Statistical Analysis

All quantitative data were shown as means ± SEM. Student’s *t*-test was used to compare the two groups. If appropriate, differences were assessed using one-way or two-way ANOVA followed by LSD test or Kruskall-Wallis tests for more than two groups as indicated. All statistical graphs were drawn using the GraphPad Prism 8.0 statistical package (San Diego, CA). Statistical analysis was performed by SPSS 23.0 (SPSS Inc, Chicago, IL, USA). A *P*-value of significance was set at *P* < 0.05.

## Supplementary Information

Below is the link to the electronic supplementary material.

Supplementary file1 (DOCX 1171 KB)

## Data Availability

No datasets were generated or analysed during the current study.

## References

[1] Ng, S.C., et al. 2018. Worldwide incidence and prevalence of inflammatory bowel disease in the 21st century: A systematic review of population-based studies. *Lancet* 390: 2769–2778. 10.1016/S0140-6736(17)32448-0 29050646

[2] Molodecky, N.A., et al. 2012. Increasing incidence and prevalence of the inflammatory bowel diseases with time, based on systematic review. *Gastroenterology* 142: 46-54 e42 (**quiz e30**). 22001864 10.1053/j.gastro.2011.10.001

[3] Sartor, R.B., and G.D. Wu. 2017. Roles for Intestinal bacteria, viruses, and fungi in pathogenesis of inflammatory bowel diseases and therapeutic approaches. *Gastroenterology* 152: 327-339 e324. 27769810 10.1053/j.gastro.2016.10.012 PMC5511756

[4] Rodriguez, C., et al. 2020. Microbiota insights in *Clostridium Difficile* infection and inflammatory bowel disease. *Gut Microbes* 12: 1725220. 32129694 10.1080/19490976.2020.1725220 PMC7524151

[5] Kudelka, M.R., et al. 2020. Intestinal epithelial glycosylation in homeostasis and gut microbiota interactions in IBD. *Nature Reviews Gastroenterology & Hepatology* 17: 597–617. 32710014 10.1038/s41575-020-0331-7 PMC8211394

[6] Lavelle, A., and H. Sokol. 2020. Gut microbiota-derived metabolites as key actors in inflammatory bowel disease. *Nature Reviews Gastroenterology & Hepatology* 17 (223): 237. 10.1038/s41575-019-0258-z 32076145

[7] Lloyd-Price, J., et al. 2019. Multi-omics of the gut microbial ecosystem in inflammatory bowel diseases. *Nature* 569: 655–662. 10.1038/s41586-019-1237-9 PMC6650278 31142855

[8] Sinha, S.R., et al. 2020. Dysbiosis-induced secondary bile acid deficiency promotes intestinal inflammation. *Cell Host & Microbe* 27 (659–670): e655. 10.1016/j.chom.2020.01.021 PMC8172352 32101703

[9] Jia, W., et al. 2018. Bile acid-microbiota crosstalk in gastrointestinal inflammation and carcinogenesis. *Nature Reviews Gastroenterology & Hepatology* 15: 111–128. 29018272 10.1038/nrgastro.2017.119 PMC5899973

[10] Stellwag, E.J., and P.B. Hylemon. 1979. 7alpha-Dehydroxylation of cholic acid and chenodeoxycholic acid by *Clostridium leptum*. *Journal of Lipid Research* 20 (325): 333. 36438

[11] Fiorucci, S., et al. 2022. Immunomodulatory functions of FXR. *Molecular and Cellular Endocrinology* 551: 111650. 10.1016/j.mce.2022.111650 35472625

[12] Bach, J.F. 2018. The hygiene hypothesis in autoimmunity: The role of pathogens and commensals. *Nature Reviews Immunology* 18: 105–120. 10.1038/nri.2017.111 29034905

[13] Maizels, R.M., and M. Yazdanbakhsh. 2003. Immune regulation by helminth parasites: cellular and molecular mechanisms. *Nature Reviews Immunology* 3 (733): 744. 10.1038/nri1183 12949497

[14] Chu, K.M., et al. 2013. Childhood helminth exposure is protective against inflammatory bowel disease: A case control study in South Africa. *Inflammatory Bowel Diseases* 19: 614–620. 10.1097/MIB.0b013e31827f27f4 23380935

[15] Shute, A., et al. 2021. Cooperation between host immunity and the gut bacteria is essential for helminth-evoked suppression of colitis. *Microbiome* 9: 186. 34517928 10.1186/s40168-021-01146-2 PMC8438845

[16] Xu, M., et al. 2018. Altered Gut microbiota composition in subjects infected with *Clonorchis sinensis*. *Frontiers in Microbiology* 9: 2292. 10.3389/fmicb.2018.02292 PMC6172334 30323795

[17] Kim, J.Y., et al. 2019. Chinese liver fluke *Clonorchis sinensis* infection changes the gut microbiome and increases probiotic *Lactobacillus* in mice. *Parasitology Research* 118: 693–699. 30623233 10.1007/s00436-018-6179-x

[18] Plieskatt, J.L., et al. 2013. Infection with the carcinogenic liver fluke *Opisthorchis viverrini* modifies intestinal and biliary microbiome. *The FASEB Journal* 27: 4572–4584. 23925654 10.1096/fj.13-232751 PMC3804743

[19] Liu, Y., et al. 2020. The role of MUC2 mucin in intestinal homeostasis and the impact of dietary components on MUC2 expression. *International Journal of Biological Macromolecules* 164: 884–891. 32707285 10.1016/j.ijbiomac.2020.07.191

[20] Winston, J.A., and C.M. Theriot. 2020. Diversification of host bile acids by members of the gut microbiota. *Gut Microbes* 11 (158): 171. 10.1080/19490976.2019.1674124 PMC7053883 31595814

[21] Robertson, S.J., et al. 2019. Comparison of co-housing and littermate methods for microbiota standardization in mouse models. *Cell Reports* 27 (1910–1919): e1912. 10.1016/j.celrep.2019.04.023 31067473

[22] Stappenbeck, T.S., and H.W. Virgin. 2016. Accounting for reciprocal host-microbiome interactions in experimental science. *Nature* 534 (191): 199. 10.1038/nature18285 27279212

[23] Khan, W.I., et al. 2002. Intestinal nematode infection ameliorates experimental colitis in mice. *Infection and Immunity* 70: 5931–5937. 12379667 10.1128/IAI.70.11.5931-5937.2002 PMC130294

[24] Yang, W., et al. 2022. GPR120 Inhibits Colitis Through Regulation of CD4(+) T Cell Interleukin 10 Production. *Gastroenterology* 162: 150–165. 34536451 10.1053/j.gastro.2021.09.018 PMC8678294

[25] Li, B., et al. 2014. IL-10 modulates DSS-induced colitis through a macrophage-ROS-NO axis. *Mucosal Immunology* 7: 869–878. 24301657 10.1038/mi.2013.103 PMC4045662

[26] Shields, V.E., and J. Cooper. 2022. Use of helminth therapy for management of ulcerative colitis and Crohn’s disease: a systematic review. *Parasitology* 149 (145): 154. 10.1017/S0031182021001670 PMC10090591 34579797

[27] Yan, C., et al. 2020. Recombinant CsHscB of carcinogenic liver fluke *Clonorchis sinensis* induces IL-10 production by binding with TLR2. *PLoS Neglected Tropical Diseases* 14: e0008643. 33044969 10.1371/journal.pntd.0008643 PMC7549790

[28] Hua, H., et al. 2021. rCsHscB derived from *Clonorchis sinensis* has therapeutic effect on dextran sodium sulfate-induced chronic ulcerative colitis in mice. *Nan Fang Yi Ke Da Xue Xue Bao* 41: 664–670. 10.12122/j.issn.1673-4254.2021.05.05 PMC8214966 34134952

[29] Xie, X., et al. 2022. Cysteine protease of *Clonorchis sinensis* alleviates DSS-induced colitis in mice. *PLoS Neglected Tropical Diseases* 16: e0010774. 36084127 10.1371/journal.pntd.0010774 PMC9491586

[30] Wang, R., et al. 2021. Gut microbiota shape the inflammatory response in mice with an epithelial defect. *Gut Microbes* 13: 1–18. 33645438 10.1080/19490976.2021.1887720 PMC7928202

[31] Shi, H., et al. 2020. Highly multiplexed spatial mapping of microbial communities. *Nature* 588: 676–681. 33268897 10.1038/s41586-020-2983-4 PMC8050837

[32] Donaldson, G.P., et al. 2016. Gut biogeography of the bacterial microbiota. *Nature Reviews Microbiology* 14: 20–32. 26499895 10.1038/nrmicro3552 PMC4837114

[33] Floudas, A., et al. 2019. *Schistosoma mansoni* worm infection regulates the intestinal microbiota and susceptibility to colitis. *Infection and Immunity* 87: e00275-e319. 31138616 10.1128/IAI.00275-19 PMC6652750

[34] Loke, P., and Y.A.L. Lim. 2015. Can helminth infection reverse microbial dysbiosis? *Trends in Parasitology* 31 (534): 535. 10.1016/j.pt.2015.10.001 PMC4752385 26604162

[35] Song, X., et al. 2020. Microbial bile acid metabolites modulate gut RORgamma(+) regulatory T cell homeostasis. *Nature* 577: 410–415. 10.1038/s41586-019-1865-0 PMC7274525 31875848

[36] McGlone, E.R., and S.R. Bloom. 2019. Bile acids and the metabolic syndrome. *Annals of Clinical Biochemistry* 56 (326): 337. 10.1177/0004563218817798 30453753

[37] Tang, W.H., et al. 2017. Gut Microbiota in cardiovascular health and disease. *Circulation Research* 120: 1183–1196. 10.1161/CIRCRESAHA.117.309715 PMC5390330 28360349

[38] Yan, C., et al. 2015. The expression dynamics of transforming growth factor-beta/Smad signaling in the liver fibrosis experimentally caused by Clonorchis sinensis. *Parasites & Vectors* 8: 70. 25649869 10.1186/s13071-015-0675-y PMC4329204

[39] Li, X., et al. 2019. H.pylori Infection alleviates acute and chronic colitis with the expansion of regulatory B Cells in mice. *Inflammation* 42: 1611–1621. 31377948 10.1007/s10753-019-01022-0

[40] Marchal-Bressenot, A., et al. 2017. Development and validation of the Nancy histological index for UC. *Gut* 66: 43–49. 26464414 10.1136/gutjnl-2015-310187

[41] Edgar, R.C. 2010. Search and clustering orders of magnitude faster than BLAST. *Bioinformatics* 26: 2460–2461. 20709691 10.1093/bioinformatics/btq461

[42] Bokulich, N.A., et al. 2013. Quality-filtering vastly improves diversity estimates from Illumina amplicon sequencing. *Nature Methods* 10: 57–59. 10.1038/nmeth.2276 PMC3531572 23202435

[43] Segata, N., et al. 2011. Metagenomic biomarker discovery and explanation. *Genome Biology* 12: R60. 21702898 10.1186/gb-2011-12-6-r60 PMC3218848

