# Supplementary material for: Clonorchis sinensis Infection prevents DSS-induced Colitis Via Lithocholic Acid in a Gut Microbiota-Dependent Manner
Source: Inflammation. 2025 Feb 27;48(5):2942–59. doi: 10.1007/s10753-025-02241-4 (PMC12596332; doi:10.1007/s10753-025-02241-4)

**Supplementary Information**

**sFigure 1 *Clonorchis sinensis* infection is established in BALB/c mice. (a)** The gross changes of livers in *C. sinensis*-infected mice on 56 days post-infection. **(b)** The change of liver index in *C. sinensis*-infected mice on 56 days post-infection. **(c~e)** The biliary injuries indicated by total bile acid (TBA, c), alkaline phosphatase (ALP, d), and total bilirubin (TBIL, e). **(f)** Histopathological changes of livers in *C. sinensis*-infected mice on 56 days post-infection. Arrow indicated worm bodies in bile duct. Compared with the indicated group, **P*<0.05, ***P*<0.01, ****P*<0.001.


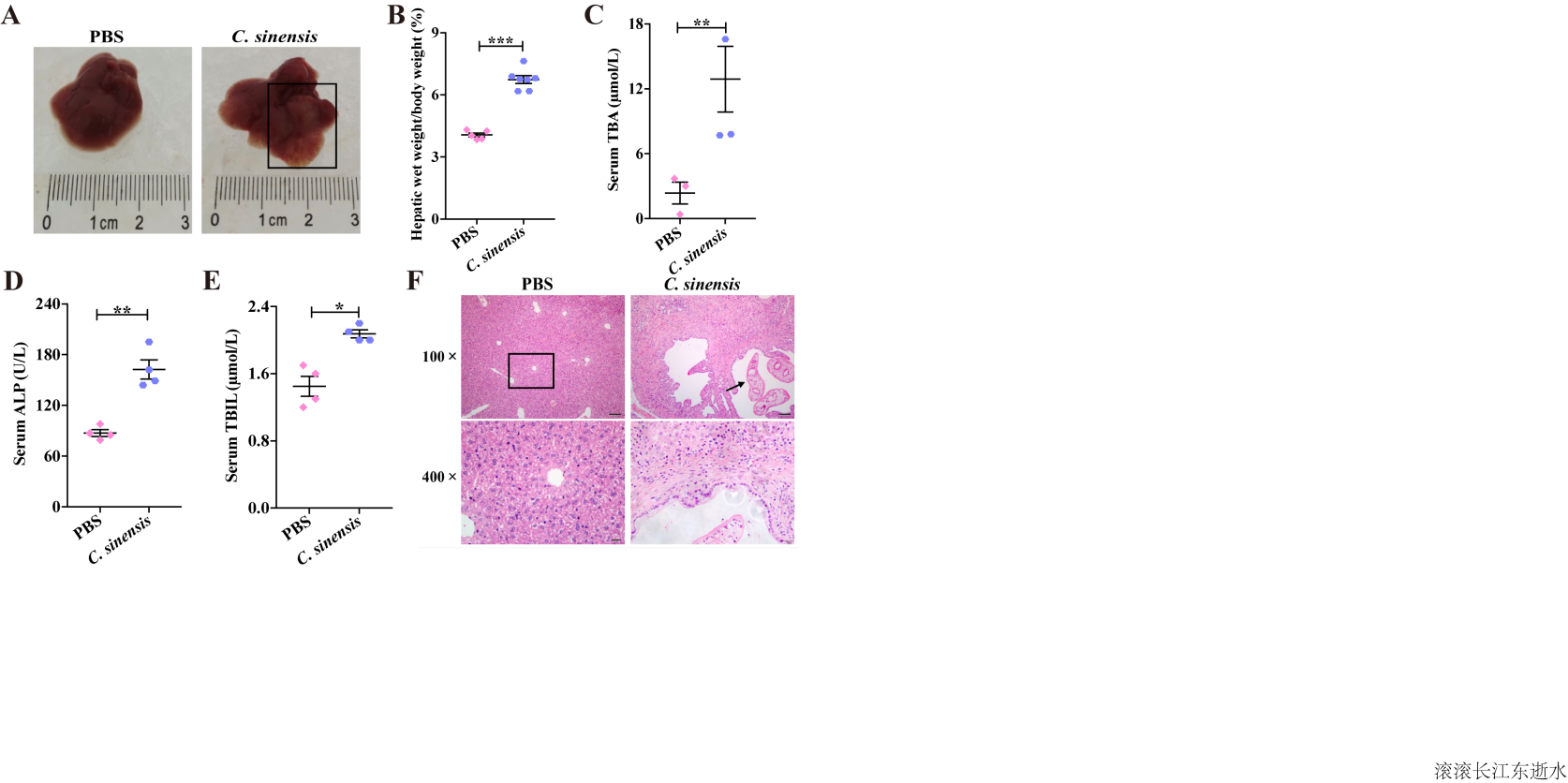


**sFigure 2 *Clonorchis sinensis* protects the gut microbiota diversity and alters the composition of gut microbiota in the DSS-induced experimental colitis. (a)** Shannon index of gut microbiota in the mice of different groups**. (b)** PCA analysis of gut microbiota in the mice of different groups. **(c&d)** The comparison of the composition of gut microbiota between PBS group and DSS treated group (**c**), DSS treated group and DSS+worm infection group (**d**). ****P*<0.001, ns means no significance.


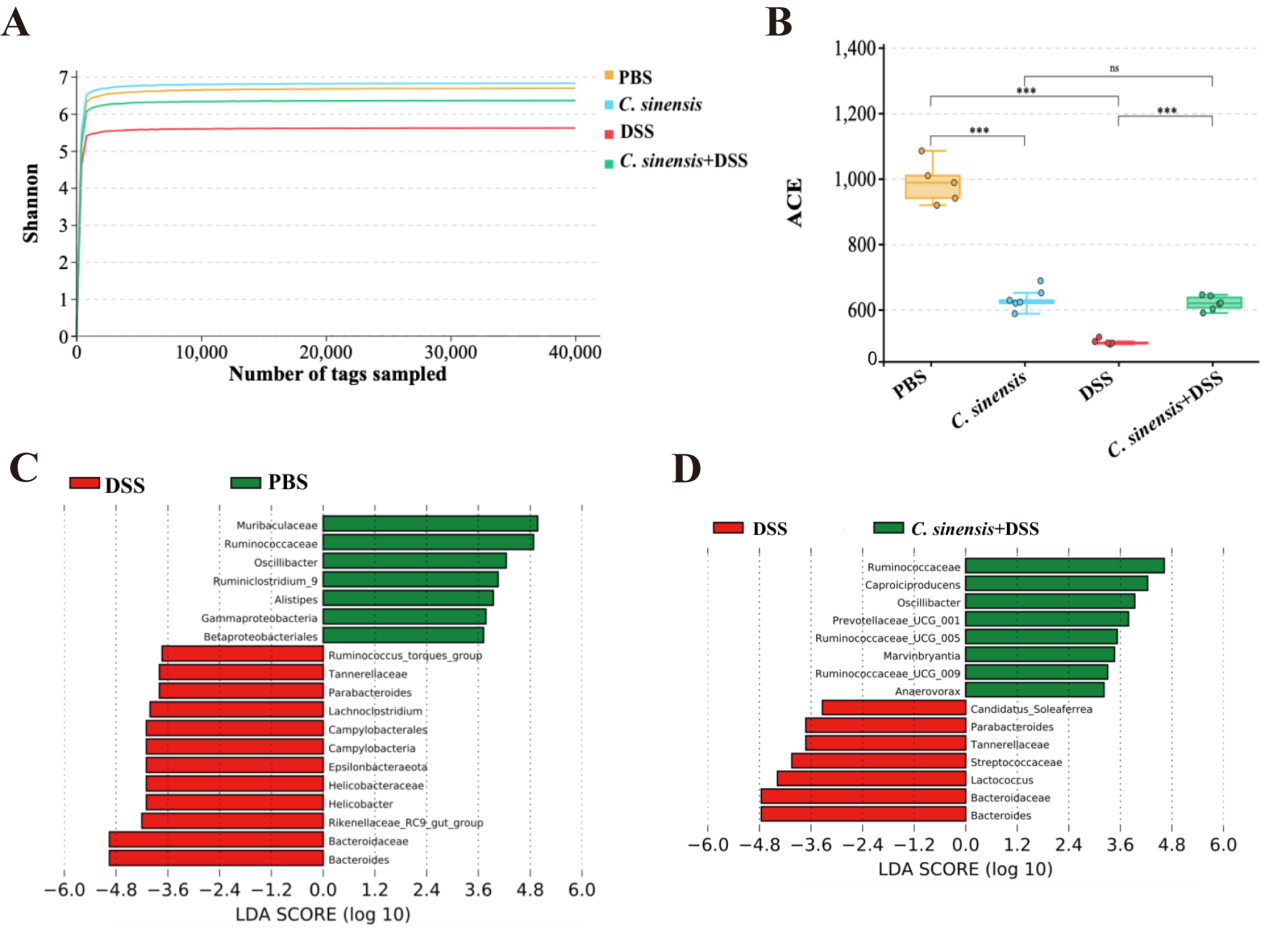


**sFigure 3 Primary bile acids and secondary bile acids in the colon of mice in each group was detected by LC-MS**.


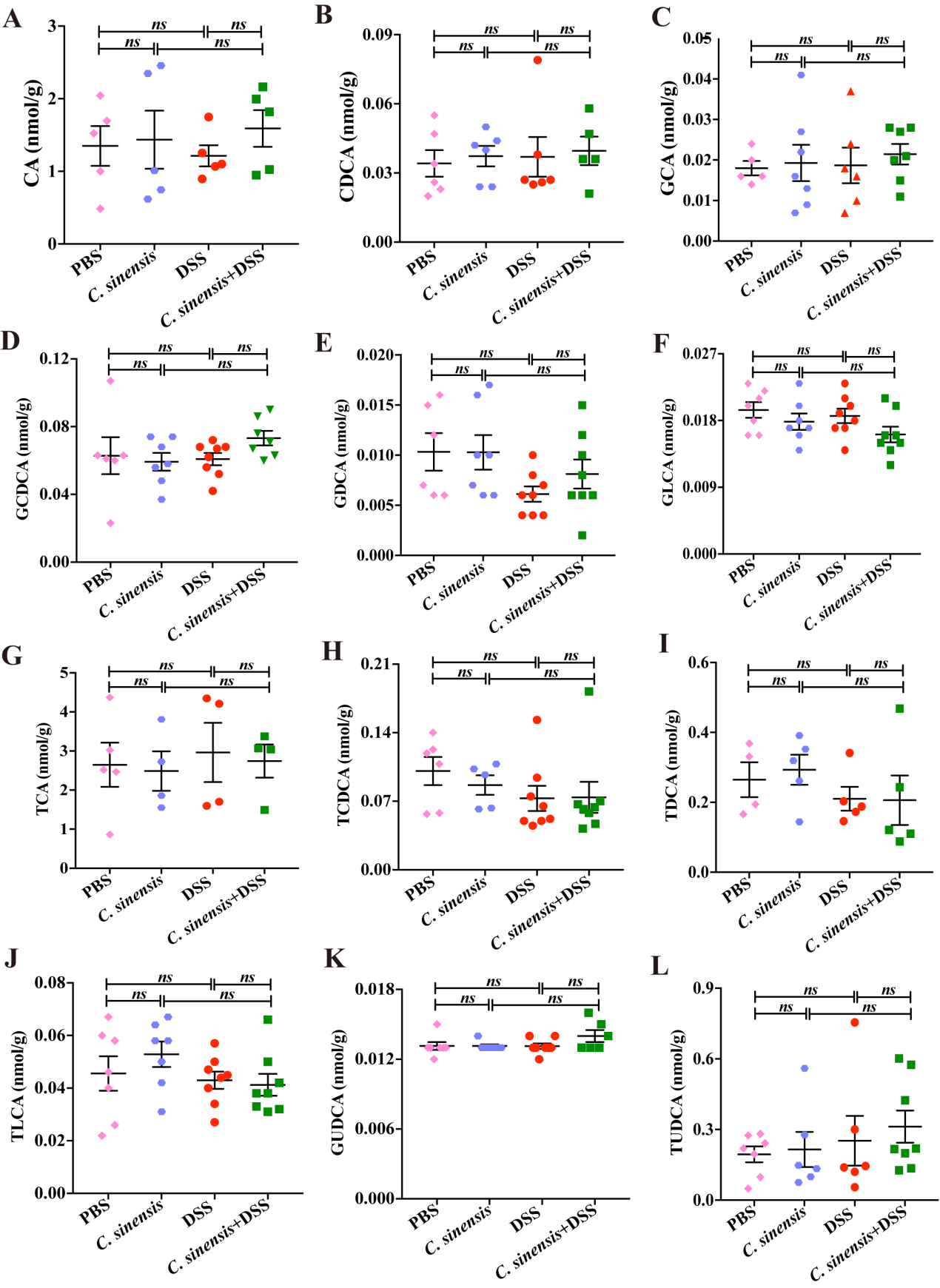


**sFigure 4 The levels of IL-10 in DSS-treated mice co-housed with *Clonorchis sinensis*-infected mice. (a)** The *Il10* mRNA in colon was detected by qPCR. **(b)** The concentration of IL-10 in colon was determined by ELISA. ns means no significance.


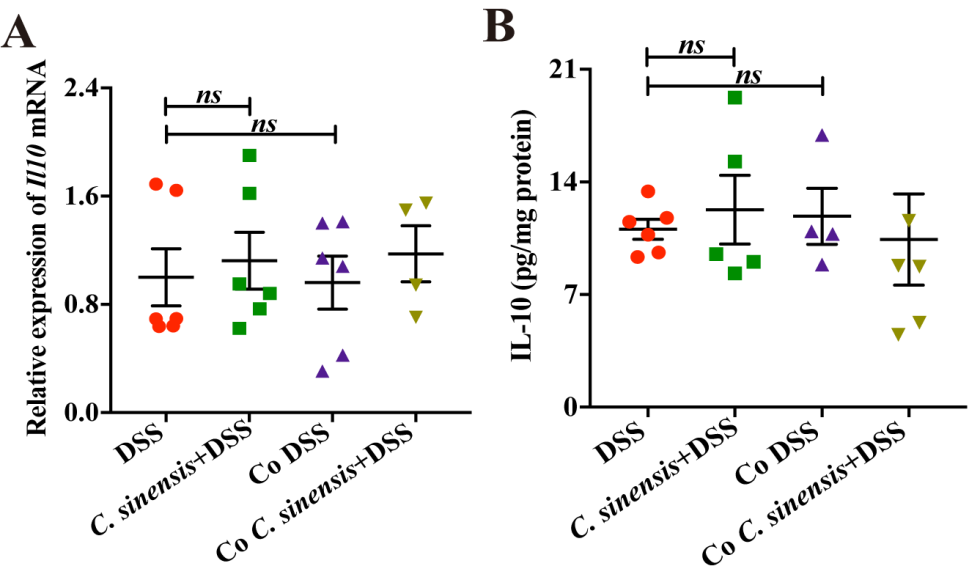

Supplement: Supplementary file 1 — Supplementary file1 (DOCX 1171 KB) [file 10753_2025_2241_MOESM1_ESM.docx]
